# Transition cow management in 27 spring-calving, pasture-based dairy herds in the south of Ireland

**DOI:** 10.3168/jdsc.2025-0890

**Published:** 2026-02-19

**Authors:** Louise Horan, Rachel Reardon, Ángel García-Muñoz, Emily M. Sitko, Ainhoa Valldecabres

**Affiliations:** 1 Teagasc, Animal & Grassland Research and Innovation Centre, Moorepark, Fermoy, Co. Cork, P61C996, Ireland; 2 School of Veterinary Medicine, University College Dublin, Belfield, Dublin D04 V1W8 Ireland; 3 Facultad de Veterinaria, Universidad Cardenal Herrera-CEU, CEU Universities, Valencia, 46115, Spain

## Abstract

In Irish spring-calving, pasture-based dairy systems, cows are typically housed during the dry period and turned out to pasture shortly after calving, unlike many other grazing systems. This study describes transition cow management practices on a convenience sample of 27 dairy farms in the south of Ireland. Farms were visited for the completion of a questionnaire and recording of housing measurements. Participants' median herd size was 142 (interquartile range = 71 to 190) cows and mean 305-d mature equivalent milk yield was 6,683 (SD = 890) kg/cow. All farms housed dry cows in ≥2 groups, grouped primarily by calving date (67%; 18/27). Ten farms (37%; 10/27) stocked cubicles above the recommended 1 cubicle per cow, and 74% (20/27) of farms had less than the recommended 0.6 m feed space per dry cow. Most farmers had calving pens (96%; 26/27), where cows were moved before signs of calving (62%; 16/26). Cow-calf separation occurred within 24 h of birth (100%; 26/26), and cows were housed for a period postcalving (100%; 26/26). Cow-level supportive health treatments, which included mainly nutritional supplements, were provided to fresh cows (64%; 16/25); calcium boluses (69%; 11/16) given to cows above a certain parity (82%; 9/11) were the most frequently used. Fresh cow health was monitored during milking (65%; 17/26), often using milk yield as an indicator (46%; 12/26). Multiple correspondence analysis and hierarchical clustering suggested that herds above the median herd size (n = 13) and producing below the mean 305-d mature equivalent milk yield (n = 16) clustered with above the recommended cubicle stocking density and below the recommended feed space per cow. Feeding practices were highly variable across farms. In conclusion, in our study sample, there was a lack of standardization in transition cow management practices suggesting that the development of recommendations tailored to this production system may be beneficial. Further, within this cohort, adequate dry cow feeding space emerged as an area warranting greater emphasis in advisory and dissemination activities.

## Highlights

• Transition period management strategies were variable between farms.
• Dry-cow feeding space warrants emphasis in dissemination activities.
• This study involves a small sample of farms in southern Ireland.

## Graphical Abstract

This study sought to describe transition cow management practices on 27 spring-calving pasture-based Irish dairy farms through a survey interview and dry-cow housing measurements. Management practices were widely variable between farms, suggesting a lack of standardization in implemented practices. Feed space was outside of recommended values on a notable proportion of farms. Multiple correspondence analysis and hierarchical clustering suggested an association between above median herd size (>142 cows) and below mean 305-day mature equivalent (ME305; <6,683 kg/cow) milk yield and overstocked dry cow housing (feed space below and cubicle stocking density above recommendation). This study provides preliminary insights into an under-reported topic on the specific dairy production system prevalent in Ireland.

**Figure:**
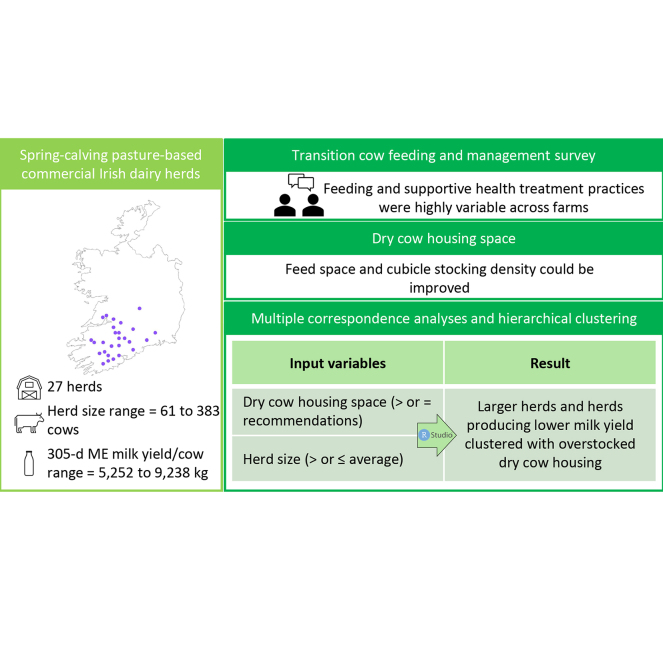

Effective management during the transition period is critical to dairy cow health, welfare, productivity, and reproductive performance, making it a key driver of sustainable dairy production. Although extensive research exists on transition cow management in housed systems, there is a notable gap in studies focused on grazing systems. Grazing systems vary across geographical locations (Roche, 2023), requiring tailored research addressing their unique challenges and opportunities. In Ireland, spring-calving, pasture-based dairy farms are most common (Shortall and Lorenzo-Arribas, 2022). Cows are typically housed during the dry period, coinciding with the winter months, and turned out to pasture shortly after calving in spring, which is in contrast with other grazing systems such as all-year-round grazing in New Zealand and Australia, but similar to grazing systems implemented in the United Kingdom and northern United States (Roche, 2023).

Assessing what practices are implemented within a specific production system is crucial for benchmarking, identifying opportunities for improvement, and setting a foundation for future research. In housed dairy production systems, surveys addressing the transition period highlight high variability of implemented management practices (Couto Serrenho et al., 2022). Kerwin et al. (2023) reported that practices such as minimizing the number of prepartum regroupings and avoiding long stays in the calving pen reduced the prevalence of elevated blood biomarkers of negative energy balance, inflammation and health disorders, and improved milk yield and reproductive performance, underscoring the importance of characterizing transition cow management and its potential role in cow performance. Previous grazing research has largely focused on evaluating individual management practices rather than surveying and describing overall transition period management. Surveys distributed online or by mail to dairy farmers in the United Kingdom and Ireland describe some reported dry (Cummins et al., 2016, n = 262; Fujiwara et al., 2018, n = 148) and transition cow management practices (Horan et al., 2024, n = 525). Studies focusing on lameness prevalence and udder health have involved herd visits and addressed some questions in relation to dry cow housing and management in Ireland (Clabby et al., 2024, n = 21; Crossley et al., 2021, n = 82; Browne et al., 2022, n = 85). The objective of this study was to describe transition cow management in 27 spring-calving, pasture-based dairy herds through a survey and on-farm observations; a study of its kind has not been previously conducted in Ireland. A recent compilation of experimental studies on grazing dairy cows has shown marked changes in mineral balance, energy metabolism, and inflammation over the transition period (Spaans et al., 2022). Knowing implemented transition cow management practices may help generate hypotheses regarding potential associations between these and markers of inflammation and metabolism, guiding future controlled research.

The current study employed a detailed questionnaire and on-farm data collection to describe transition cow management practices on a convenience sample of 27 dairy farms in the south of Ireland. Participants were selected from 119 respondents to an online transition period survey addressed to Teagasc Technical Dairy Advisory clients (Horan et al., 2024) who met the following eligibility criteria: (1) provided consent to be contacted about the study, (2) Irish Cattle Breeding Federation (**ICBF**) client with an active HerdPlus subscription, (3) provided consent for Teagasc to access their ICBF profile, (4) spring-calving herd, (5) milking herd size >60 cows, (6) located within a 2 h drive from Teagasc Moorepark (Fermoy, Co. Cork, Ireland), and (7) minimum of 4 milk recordings completed in 2022. The sample was chosen using stratified random sampling according to herd mean 305-d mature equivalent (**ME305**) milk yield quartiles and reported annual bought-in feed per cow (≤ or >1 t). All data required for eligibility verification and stratification were obtained from the online survey except herd size and ME305 milk yield, which were collected from farmers' ICBF profiles.

A questionnaire was completed through an oral interview during a herd visit in early spring 2023. The questionnaire addressed dry and fresh period feeding and management practices through 25 questions and was designed using SurveyMonkey (SurveyMonkey Inc., San Mateo, CA). Dry-off-related questions that have been previously reported by others are not discussed herein; results of 23 questions are discussed instead. All questions were kept open-ended, and results were recorded on paper during the interview. The total number of cubicles available to dry cows across housing groups was counted and total feed space was measured using a 30.48-cm measuring wheel (Dargan Tools Ltd. Co., Carlow, Ireland). Collected data were transferred to an Excel spreadsheet (Microsoft Corp., Redmond, WA) for cleaning and summarization. Similar and infrequent responses were grouped into broader categories (e.g., dry cow feeding frequencies “every second day” and “every third day” became “less frequently”). Due to a questionnaire implementation error, fresh period questions were completed by 26 farmers, whereas the dry period questions were completed by all 27 farmers. Denominators are provided in the text to show where a question may have been missed or not answered by some participants, or where results were contingent on responses to prior questions.

Multiple correspondence analysis (**MCA**), an exploratory, descriptive, and noninferential method to investigate relationships among categorical variables by dimension reduction (Greenacre, 2017), was performed using RStudio (version 4.4.2; https://www.r-project.org/). A dataset including only the most objective variables available: the directly measured data (feed space and cubicle stocking density) and variables obtained from ICBF (herd size and mean ME305 milk yield), was used to visualize associations between study-measured variables and herd demographic characteristics by MCA using the FactoMineR package (Lê et al., 2008). The chosen number of variables ensures stability and interpretability of the results, as they represent no more than one-fifth the number of variables relative to participants (Greenacre, 2017). Categories for milking herd size and herd mean ME305 milk yield were “above average” and “below average” (median milking herd size = 142 cows, mean ME305 milk yield = 6,683 kg/cow), and categories for feed space and cubicle stocking density were “as per recommendation” and “above recommendation” (cubicle stocking density: ≥1 cubicle per cow [EFSA Panel on Animal Health and Animal Welfare et al., 2023a]; feed space: 0.6 m/cow [Rioja-Lang et al., 2012]). Hierarchical clustering using the Ward.D2 method (Ward, 1963) was implemented on the MCA coordinates from the first 2 dimensions with the *hclust* function from the stats package (R Core Team, 2024). After visual inspection of the relative heights of linkage distances at which clusters merged, the resulting dendrogram was cut manually to yield 2 clusters. Coordinates from the MCA and ellipses representing the distribution of coordinates within each cluster were plotted in 2 dimensions using ggplot2 (Wickham, 2016).

Participants' median milking herd size was 142 (interquartile range = 71 to 190) cows, mean (± SD) ME305 milk yield was 6,683 ± 890 kg/cow, and mean (± SD) calving interval was 369 ± 7 d. Dry cows were housed in covered cubicle pens on all study farms, except one, where they were housed in uncovered cubicle pens. Similarly, a survey of 21 Irish dairy herds reported that all herds housed cows in covered cubicle pens (Clabby et al., 2024). Dry cows were housed in either >2 (63%; 17/27) or 2 (37%; 10/27) groups, with grouping decisions based mainly on expected calving date (67%; 18/27) and parity (30%; 8/27), with 2 farmers reporting grouping based on BCS and 3 farmers reporting no specific criteria. Surveys conducted in housed dairy production systems in the United Kingdom (n = 148) and Canada (n = 78) report grouping dry cows in 2 groups by expected calving date on the majority of farms (73% and 81%, respectively; Fujiwara et al., 2018; Couto Serrenho et al., 2022). Cows were regrouped more than once (41%; 11/27), once (33%; 9/27), or not at all (26%; 7/27). Regrouping can promote negative interactions among housed dry cows and reduce DMI, especially for primiparous cows (Proudfoot and Huzzey, 2022). Across groups, farmers reported providing different diets (59%; 16/27), increased observation (e.g., close-up cows; 26%; 7/27), or to not vary management (30%; 8/27). Grouping cows by calving date allows for targeted nutritional management to prevent metabolic disorders (Rissanen et al., 2024) and facilitates observation of close-up cows for identification of calving or health problems.

Dry cow housing provided between 0.4 and 1.5 cubicles per cow across the different housing groups. At least 1 cubicle per dry cow was available in 63% of the farms (17/27), as recommended by the European Food Safety Authority (EFSA Panel on Animal Health and Animal Welfare et al., 2023a). The remaining 37% (10/27) of farms stocked cubicles above this recommendation, notably less than the 76% of Irish pasture-based farms above this recommendation reported by Clabby et al. (2024). Overcrowding in housed dairy cow cubicles has been associated with decreased lying and ruminating (Jiang et al., 2021), indicating lower cow comfort and feed intake. Feed space per cow across the different housing groups ranged between 0.2 to 0.7 m across farms, with 74% (20/27) of farms not meeting the recommendation of 0.6 m/cow (Rioja-Lang et al., 2012), comparable to the 58% of Irish pasture-based farms reported by Browne et al. (2022) for the same. Limited feeding space may compromise cows' access to top-dressed minerals, as commonly provided in the study farms (78%; Horan et al., 2025). Optimizing feed space per dry cow in housed dairy production has been associated with a reduced prevalence of elevated postpartum energy metabolites and inflammatory markers, minimized health disorder incidences, and maximized milk yield and improved reproductive success in the subsequent lactation (Kerwin et al., 2023). By measuring stocking density at the overall housing level (across the different housing groups), we were unable to identify potential within-housing group variation.

Pasture silage delivered once daily (59%; 16/27), less frequently (37%; 10/27), or more frequently (4%; 1/27) was the primary forage offered throughout the dry period on all farms. Increased feed delivery frequency has been associated with increased feeding time and fewer displacements of subordinate cows from the feed space in housed lactating dairy cows (DeVries et al., 2005). Whereas 44% (12/27) of farmers fed pasture silage alone, 56% (15/27) provided additional feed sources throughout the dry period, which included concentrates (53%; 8/15), barley straw (27%; 4/15), and maize meal (20%; 3/15). Similarly, Fujiwara et al., (2018) reported 68% and 65% of respondents providing close-up housed dry cows with straw and concentrates or other source of protein, respectively. Nevertheless, a recent study suggests that supplementation of housed grass silage-fed dry cows with concentrates has no effect on feed intake, energy metabolism, or subsequent lactation performance (Rissanen et al., 2024).

A plot showing the MCA and hierarchical clustering results is presented in Figure 1. The first 2 dimensions of the analysis accounted for 56% of the total inertia (dimension 1: 36%; dimension 2: 20%). Hierarchical clustering revealed 2 clusters within this dataset; farms with a milking herd size above the median and farms with a ME305 milk yield below the mean were clustered with feed space below recommendation and cubicle stocking density above the recommendation. On the contrary, farms with a milking herd size below the median and farms with a ME305 milk yield above the mean were clustered with feed space as per recommendation and cubicle stocking density as per recommendation. Results from this dataset may reflect the negative association between dry cow housing stocking density, dry cow rumination, and milk yield described by Jiang et al. (2021) for housed dairy cows, and align with the suggestion by Schütz et al. (2025) that rapid herd expansion in the years preceding European Union milk quota abolishment coupled with inadequate housing investment in the years before abolishment has resulted in suboptimal housing on pasture-based farms.

**Figure 1:**
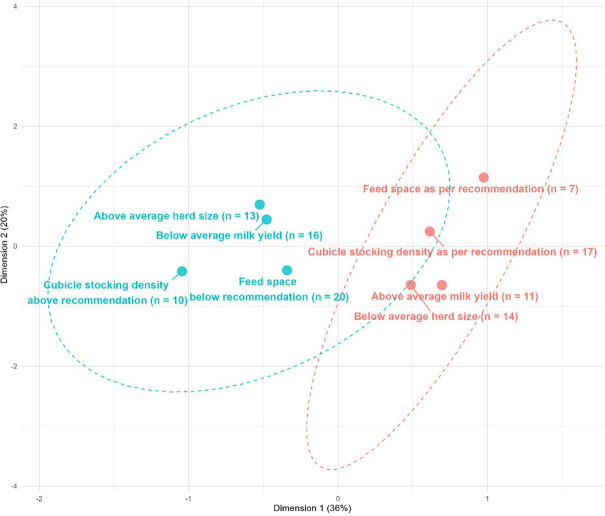
Map of multiple correspondence analysis and hierarchical clustering for the association between herd demographics (milking herd size [median = 142 cows] and herd mean 305-d mature equivalent milk yield [mean = 6,683 kg/cow]) and study-measured variables (dry cow feeding space [recommendation 0.6 m/cow; Rioja-Lang et al., 2012] and cubicle stocking density [recommendation ≥1 cubicle per cow; EFSA Panel on Animal Health and Animal Welfare et al., 2023a]). The number of farms in each category and the proportion of inertia are presented in parentheses.

Almost half of farmers had both group and individual calving pens (46%; 12/26), 35% (9/26) had individual pens only, 15% (4/26) had group pens only, and 4% (1/26) had none. In contrast, group calving pens were most commonly used by Irish dairy farmer respondents (49%) reported by Cummins et al. (2016). The use of individual calving pens may reduce the transmission of certain diseases; however, from a welfare and behavior viewpoint, a group calving pen with a secluded area does not disrupt cows' natural herd instincts before calving and allows them to perform natural isolation behaviors at calving (Proudfoot et al., 2014). Farmers moved cows into calving pens before signs of calving were observed (62%; 16/26). Similarly, Cummins et al. (2016) reported 41% of respondents moved cows >24 h precalving, in line with suggestions to help avoid prolonged calvings and elevated inflammation (Proudfoot et al., 2013). Among farmers moving cows once calving signs were observed (19%; 5/26), 3 moved cows once in active labor and 2 when early signs, such as a raised tail, were noticed. Other farmers indicated moving cows before signs of calving during the day but only when signs of calving were observed in the night (15%; 4/26). Farmers using calving pens and answering a question related to feeds provided in them indicated that pasture silage and water were supplied (24/24); some of these farmers also provided concentrates (21%; 5/24), minerals (i.e., powdered fresh cow minerals, dry cow minerals, calcium and magnesium combined supplement, or magnesium flakes; 16%; 4/24), or concentrates and minerals fed together (4%; 1/24). Providing feeds that fill the rumen easily postpartum (e.g., forages) helps reduce the risk of displaced abomasum (Van Winden et al., 2003). Only 1 farmer reported using an automated tail movement sensor device to predict calving time. In all farms (26/26), calves were separated from the dam within 24 h of birth, which, although it is globally a common practice (Couto Serrenho et al., 2022), is in contrast with the European Food Safety Authority's recommendation of allowing cow-calf contact for at least 24 h postcalving (EFSA Panel on Animal Health and Animal Welfare et al., 2023b). Farmers separated calves at regular times throughout the day (35%; 9/26), after some cow-calf contact (i.e., suckling or maternal grooming; 35%; 9/26), immediately upon observing the calving had occurred (23%; 6/26), or depending on timing of calving (day or night; 8%; 2/26).

Farmers kept cows housed for ≤1 wk postcalving (58%; 15/26), with 27% (7/26) reporting that the decision was weather dependent. Two farmers did not house cows postcalving (8%; 2/26), one farmer housed cows until they expelled their placenta (4%; 1/26), and another farmer housed all cows for 1 mo postcalving (4%; 1/26). After turnout, farmers brought cows indoors at night for a period postcalving (76%; 19/25), 20% (5/25) of farmers reported that this practice was weather dependent, and 1 farmer did not implement this practice (4%; 1/25). Cows were under this night housing management practice until March or beginning of April (53%; 10/19), for between 10 d and 6 wk after the block calving start date (26%; 5/19), or for a set number of days or weeks that varies each year (21%; 4/19). During the postcalving housing period, fresh cows were kept in the dry cow housing (75%; 18/24). Only 5 farms had specific housing for fresh cows (21%; 5/24) and 1 farmer reported keeping some cows in fresh cow housing and some in the dry cow housing (4%; 1/24). Overall, weather conditions were a key consideration in deciding when to house cows' postpartum. Inclement weather can increase the risk of some metabolic diseases (Roche and Berry, 2006).

Farmers milked cows twice a day throughout their entire lactation (65%; 17/26), which is the most common milking frequency adopted in Ireland (Uí Chearbhaill et al., 2024). The remaining farmers milked cows once a day postcalving (35%; 9/26) and were doing so for anytime between 4 d to 4 wk from the block calving start date (78%; 7/9) before adopting twice-a-day milking for the remainder of the cows' lactations. Milking grazing dairy cows once a day for 3 or 6 wk after calving can negatively affect lactation milk yield and milk component yield, but can improve cow energy status and BCS (Phyn et al., 2014).

Concentrates were provided during milking on all farms (26/26) and feeding rates varied from 1.5 to 6.5 kg/cow per day. Fresh cow minerals were provided by 19% (5/26) of farms; 4 farmers provided them in powder form and 1 through the water troughs. Most herds grazed pasture and provided pasture silage in the fresh period (96%; 25/26), except for the single herd where cows were housed for 1 mo postcalving, and therefore only pasture silage was provided to fresh cows. All herds therefore provided pasture silage to fresh cows; however, Walsh et al. (2023) suggests milk production benefits from increasing fresh pasture and decreasing pasture silage in the early lactation diet of Irish pasture-based dairy cows. Farmers reported measuring pasture and allocating it accordingly to cows (84%; 21/25). Some farmers added other ingredients to the fresh cow diet when cows were housed, mainly beet (12%; 3/26), barley straw (12%; 3/26), and maize (12%; 3/26), and the remainder did not add any of these ingredients (65%; 17/26).

Cow-level supportive health treatments, which included mainly nutritional supplements, were provided to fresh cows (64%; 16/25). Of the farmers providing prophylactic treatments, oral calcium boluses were most common (69%; 11/16). Farmers reported multiple criteria for providing calcium boluses, although precise details of criteria implementation were not asked: mainly for cows above a certain parity (82%; 9/11) or BCS (36%; 4/11), but also for cows with late calving, dystocia, or milk fever history (each 1/11). In our recent online survey, 18% of the farmers reported supplementing calcium postpartum (88/487; Horan et al., 2024). Farmers also reported providing drinks (25%; 4/16), oral drenches (19%; 3/16), and s.c. calcium (13%; 2/16) aimed at preventing metabolic disorders, as well as a dose of anthelmintic, limestone, yeast bolus, nonsteroidal anti-inflammatory, and i.v. calcium (each 1/16).

Cows' health was mainly monitored during milking (65%; 17/26), after milking (35%; 9/26), or by activity or rumination monitors (31%; 8/26). Methods used for monitoring cows' health mainly included milk yield and composition surveillance (81%; 21/26). A smaller proportion of farmers reported performing blood mineral testing (23%; 6/26), locomotion scoring (19%; 5/26), BCS (12%; 3/26), and concentrate intake monitoring (8%; 2/26), whereas 2 farmers reported that they do not systematically monitor cows' health. Methods used were in agreement with recommended approaches for metabolic disease detection (Mulligan et al., 2006). Almost half of the farmers reported having herd health issues postcalving in previous years (46%; 12/26), with milk fever being the most common (50%; 6/12), followed by retained placenta (12%; 3/12), left displaced abomasum (12%; 3/12), pneumonia (8%; 2/12), as well as ketosis, iodine deficiency, and phosphorus deficiency (each 1/12). Hypocalcemia has been reported to be one of the most common metabolic conditions of grazing dairy cows around calving (Spaans et al., 2022; Horan et al., 2024).

A limitation of this study is the convenient sample of 27 farms, which is small relative to the total number of dairies in Ireland and drawn from a defined subgroup of farmers (Teagasc Technical Dairy Advisory clients located in the south of the Republic of Ireland). Thus, we warn readers of the potentially limited generalizability of our results and underscore the effect that a small change in the number of responses could have had on the trends reported as most frequent results in this study. In conclusion, implemented transition period management practices varied considerably across the farms in this dataset, suggesting a lack of standardization in current practices, which could decrease if specific recommendations arise from transition cow research tailored to this production system. Overstocking of dry cow housing was observed on several study farms; thus, this area may warrant greater emphasis in advisory and dissemination activities. This study provides preliminary insights into an under-reported spring-calving, pasture-based dairy production system.
